# Thermal Shock and Oxidation Behavior of HiPIMS TiAlN Coatings Grown on Ti-48Al-2Cr-2Nb Intermetallic Alloy

**DOI:** 10.3390/ma9120961

**Published:** 2016-11-25

**Authors:** Claudio Badini, Silvia M. Deambrosis, Elisa Padovano, Monica Fabrizio, Oxana Ostrovskaya, Enrico Miorin, Giuseppe C. D’Amico, Francesco Montagner, Sara Biamino, Valentina Zin

**Affiliations:** 1 Department of Applied Science and Technology, Politecnico di Torino, Corso Duca degli Abruzzi 24, Torino 10129, Italy; elisa.padovano@polito.it (E.P.); oxana.ostrovskaya@polito.it (O.O.); d027201@polito.it (G.C.D.); sara.biamino@polito.it (S.B.); 2 National Research Council (CNR) of Italy, Istituto di Chimica della Materia Condensata e di Tecnologie per l’Energia (ICMATE), Corso Stati Uniti 4, Padova 35127, Italy; silviamaria.deambrosis@cnr.it (S.M.D.); monica.fabrizio@cnr.it (M.F.); enrico.miorin@cnr.it (E.M.); f.montagner@ieni.cnr.it (F.M.); valentina.zin@cnr.it (V.Z.)

**Keywords:** HiPIMS TiAlN coating, burner rig, oxidation behavior, thermal shock resistance

## Abstract

A High Power Impulse Magnetron Sputtering (HiPIMS) method for depositing TiAlN environmental barrier coatings on the surface of Ti-48Al-2Cr-2Nb alloy was developed in view of their exploitation in turbine engines. Three differently engineered TiAlN films were processed and their performance compared. Bare intermetallic alloy coupons and coated specimens were submitted to thermal cycling under oxidizing atmosphere up to 850 °C or 950 °C, at high heating and cooling rates. For this purpose, a burner rig able to simulate the operating conditions of the different stages of turbine engines was used. Microstructures of the samples were compared before and after each test using several techniques (microscopy, XRD, and XPS). Coating-intermetallic substrate adhesion and tribological properties were investigated too. All the TiAlN films provided a remarkable increase in oxidation resistance. Good adhesion properties were observed even after repeated thermal shocks. HiPIMS pretreatments of the substrate surfaces performed before the coating deposition significantly affected the oxidation rate, the oxide layer composition and the coating/substrate adhesion.

## 1. Introduction

Titanium aluminides have been widely investigated in the last two decades because of their potential for high temperature aerospace and automotive applications [1,2,3]. In fact, these materials display attractive properties such as low density, high specific stress, high specific stiffness and good corrosion, creep and oxidation resistance. On the other hand, it is well known that their lack of room temperature ductility and fracture toughness has represented the main cause of concern for industrial applications. During recent years, better knowledge of their microstructure and its effect on the deformation mechanism, the progress in manufacturing technologies, the optimization of thermal treatments and the development of new alloys have led to a commercial use of titanium aluminides for turbochargers of cars and gas turbine blades [4,5,6,7,8]. For instance, General Electric is currently using Ti-48Al-2Cr-2Nb intermetallic alloy in the low pressure turbine blades of its GEnex engine designed for the Boeing 787 Dreamliner [6]. On the one hand, the adoption of titanium aluminides in the aeronautic field as an alternative to nickel-base superalloys would allow for significant weight reduction of the structural components of gas turbines (20%–30%). On the other hand, the envisaged replacement of superalloys would require the development of titanium aluminides matching the performance of Ni-based superalloys, also at very high temperatures. The Ti-48Al-2Cr-2Nb alloy is currently used for low pressure turbine blades operating at around 750 °C, but new intermetallics containing Nb, Si and Mo have shown improved oxidation, creep and high-temperature fatigue resistance and are presently under investigation [9,10]. These TiAl alloys of second and third generation show specific strength over Ni-base alloys at temperatures up to 800 °C. The third generation alloys can be used up to 850 °C. Moreover, it is expected that low pressure turbine blades will be required to run faster and at hotter in more efficient future engines. Further alloying addition is presently being investigated with the objective of using new TiAl alloys at even higher operating temperatures (900–1000 °C) [11]. For such envisaged applications, the oxidation resistance of the turbine components is of great importance. Oxidation resistance should be displayed under particular environmental conditions since inside a turbine the atmosphere contains not only oxygen but water vapor and nitrogen, too. In the past, thermal barrier coatings (TBCs) [12] greatly contributed to increasing the maximum operating temperature of turbine engines since they provided reliable oxidation protection to hot structural parts. However, TBCs showed some drawbacks (like residual stress arising from thermal mismatches between their components and the substrate) that can cause coating failure [13]. In view of a wider adoption of titanium aluminides in turbine engines, protective coatings specifically designed for this application should also be developed. TiAlN seems very promising for titanium aluminide protection. First of all, its thermal expansion coefficient is equal to 7.5 × 10^−6^ K^−1^ [14] and not very far from that of Ti-48Al-2Cr-2Nb (10 × 10^−6^ K^−1^ according to our measurements). Moreover, their chemical compositions are similar enough to promote adhesion. In addition, TiAlN coatings should grant very good wear and oxidation resistance [15,16,17,18]. Several methods have been proposed for the deposition of TiAlN coatings on many different substrates, like steel, silicon, glass and alumina [15,16,19,20,21,22]. Only a few investigations have concerned the deposition of TiAlN on titanium aluminides by arc ion plating [23] or by magnetron sputtering [24]. To our knowledge, High Power Impulse Magnetron Sputtering (HiPIMS) has never been used for titanium aluminum nitride deposition on ϒ-TiAl based substrates.

In this research work, reactive HiPIMS technology [25,26,27,28,29] was employed to process TiAlN coatings for Ti-48Al-2Cr-2Nb protection. The advantages of the sputtering technique are the uniform distribution of the elements, the possibility to adjust the chemical composition of the deposited layer in a wide range and excellent surface quality. Small modifications of its basic principle are used in order to deposit compounds (i.e., oxides or nitrides) via reactive sputtering [29,30]. Reactive sputtering is carried out by introducing into the process chamber a gas or a mixture of gases that reacts with the target material to form a coating of a different chemical composition. The basis of HiPIMS is to increase the plasma density in front of a sputtering source in order to decrease the mean ionization distance for the sputtered particles. The increase in plasma density is simply achieved by applying a high electrical power in pulses with a low duty factor (low ratio pulse on time/cycle time) [31]. Therefore, HIPIMS is a simple method that combines the advantages of conventional magnetron sputtering and arc evaporation, since it produces a highly ionized droplet-free plasma. 

With respect to traditional magnetron sputtering techniques, HiPIMS plasma conditions greatly enhance the flux of energetic ionized species towards the growing film, allowing smoother and denser films to be deposited on complex-shaped substrates. In addition, by tuning the HiPIMS process parameters, novel structures and an increased ratio of hardness to elastic modulus can be achieved [32]. Moreover, the highly ionized conditions in HiPIMS are also well suited for a material pretreatment aimed at the removal of the natural oxide layer that exists on most materials [33] and at adhesion improving [34]. Indeed, in conventional pretreatment, the substrate is bombarded with high-energy gas ions, usually Ar ions. In HiPIMS, a fraction of the plasma ions comes from the target material. For this reason, during the etching process some of the bombarding metal ions may be implanted into the interface region, creating a gradual change in the composition between the substrate and the film. This feature is believed to have beneficial effects on the adhesion of the film. In this paper, a HiPIMS method suitable for the deposition of TiAlN coatings on the surface of Ti-48Al-2Cr-2Nb alloy is presented. The effect of processing parameters on the coating performance was investigated. The behavior of the coatings was tested using a burner rig specifically designed for simulating the environmental conditions faced by hot structural components of gas turbines. The detrimental effect, which may occur during ageing because of temperature, oxidation and thermal shocks, is discussed.

## 2. Materials and Methods 

Bars of Ti-48Al-2Cr-2Nb alloy with a diameter of 25 mm were processed by electron beam melting and then thermally treated; the processing method has been detailed in a previous paper [8]. Cylindrical specimens (20 mm high) were cut from these bars and their upper surfaces were metallographically polished to a 1 µm diamond finishing. 

All the TiAlN coatings were deposited using a unique set of working parameters (shown in Table 1) by reactive HiPIMS (TRUMPF-Hüttinger, True Plasma High Pulse 4002 power supply and 18 kW model 3018 HBP bias unit, Freiburg, Germany). The chosen target (Ti_0.5_Al_0.5_ 99.9% purity) was mounted on a weakly unbalanced magnetron cathode. The substrates, placed 100 mm below the target, were heated up to 400 °C and maintained at this temperature throughout the process by additional heaters and thermocouple monitoring. The turbo-molecular pumped high-vacuum chamber was evacuated prior to the depositions to a base pressure lower than 1 × 10^−7^ mbar. Both the inert gas (Ar, 99.9997% purity) and the reactive gas (N, 99.998% purity) were introduced through dedicated mass flow controllers. 

Three different sets of specimens were deposited. Based on preliminary results, a single set of optimized working parameters (shown in Table 1) was used to deposit all the TiAlN samples. Instead, the surface preparation of the Ti-48Al-2Cr-2Nb substrate alloy was modified in an attempt to affect the growing mode (see Table 1). Films TiAlN1 (three identical samples in one sputtering run) were obtained without any specific surface treatment; only ultrasound cleaning was performed before the coating deposition. For the three equivalent specimens named TiAlN2, in addition to ultrasound cleaning, a 60 min plasma etching was carried out (Ti_0.5_Al_0.5_ cathode average power 100 W, bias voltage −1200 V, Ar pressure 1.0 × 10^−2^ mbar, frequency 500 Hz, and pulse length 25 μs). Finally, samples labeled TiAlN3 were equal to TiAlN2 films, except for the pretreatment duration (10 min) and the interposition of a metal layer (TiAl) sputtered for 10 min without introducing nitrogen in the chamber.

The uncoated substrates together with the coated specimens were submitted to the same oxidation treatments. A burner rig apparatus, specifically designed to test the hot structural components of turbines, was used for this purpose. The specimens were heated by a flame fed by methane and air, using air in excess (17:1 air to fuel volume ratio). The burner was fed by using calibrated mass flow controllers for oxygen and methane. The oxygen content in the exhausts was calculated assuming that complete combustion occurred. They were submitted to 40 thermal cycles, each of them consisting of: a quick heating step up to a pre-fixed maximum temperature (850 °C or 950 °C, heating rate 141 °C/min), an isothermal step of three minutes at the maximum temperature and a final step of fast air-quench down to 300 °C (cooling rate 185 °C/min). During each cycle, the oxygen concentration firstly progressively increased from 3.1 to 8.7 vol %, it remained constant during the isothermal step (8.7 vol %) and then suddenly increased up to 21 vol % during the quench. Thermal cycles were computer controlled through thermocouples placed closed to the sample surfaces.

The effect of thermal shocks and oxidation was investigated using several techniques. Morphologies and microstructures of top surfaces and cross sections were studied by microscopy (Leica DMI 5000 M optical microscope, SEM-FEG Assing SUPRA 25, Oberkochen, Germany, equipped with EDS Oxford INCA X-sight). X-ray diffraction was used to identify crystalline phases (Panalytical X’PERT PRO PW3040/60, Panalytical BV, Almelo, The Netherlands, Cu Kα radiation; Micro-XRD Rigaku D/max probe 100 micron). TiAlN films and substrate were analyzed by X-ray Photoelectron Spectrometry (XPS, PHI 5000 Versa Probe, Chanhassen, MN, USA), before and after different burner rig tests. XPS analyses were repeated after sputtering (accelerating voltage 2 kV, 15 mPa partial pressure, area 2 mm × 2 mm) the sample surface in order to get information about the oxide thickness and the chemical composition versus depth. All the samples were sonicated in an alcohol bath before the analyses. In addition, before each XPS investigation, the sample surface was sputtered step by step (sputtering conditions as reported above, step of 1 min) by argon ions until the C1s peak intensity became negligible (less than 1 at %). Monochromatic Al Kα, 1486.6 eV energy, 15 kV voltage and 1 mA anode current were used for the XPS analysis. A spot size of 100 µm was used to collect the photoelectron signal for both the survey and the high resolution (HR) spectra. An electron beam source was used in order to compensate the surface positive charging phenomenon. Different pass energy values were employed: 187.85 eV for survey spectra and 23.5 eV for HR peaks. Calibration of XPS equipment was performed by matching the literature binding energy values of Au 4f_7/2_, Cu 2P_3/2_ and Ag 3d_5/2_ peaks. The spectra were analyzed (for deconvolution and fitting) through Multipak 9.6 software (Physical Electronics Inc., Chanhassen, MN, USA). 

Adhesion and tribological properties of the TiAlN/Ti-48Al-2Cr-2Nb systems were investigated with scratch tests by using an UMT-2 tribotester (CETR now Bruker, Billerica, MA, USA), equipped with a standard Rockwell C diamond tip or alumina balls. Scratch test parameters were chosen according to ISO 1071-3:2005 standard [35] for ceramic materials in progressive loading scratch test (PLST) mode. The normal force (F_z_) and the friction coefficient (COF) were recorded as a function of time. The coating/substrate adhesion was quantified determining the failure critical load Lc3, at which adhesive failure was initiated due to the coating delamination. Lc3 was estimated by combining detected signals with microscope images of scratch tracks (Wild/Leica M3Z stereomicroscope equipped with a Lumenera Infinity lite camera). Dry sliding tests in a ball-on-flat configuration were performed using an UMT-2 tribotester in order to evaluate the wear resistance. A 5 mm diameter alumina ball was employed as a counter-body. Wear tests were conducted for 1700 cycles at a speed of 10 mm·s^−1^ by applying a load of 1.2 N in order to obtain a Hertzian contact pressure of ~1 GPa on coupled surfaces, thanks to a non-conformal contact type. The topography of the worn surfaces was examined by microscopy and stylus profiler (Bruker Dektak XT equipped with a 2 μm tip). The worn volume was calculated according to ASTM G133-02 [36] and wear rate was evaluated as well [37].

## 3. Results

### 3.1. Microstructure Investigation

**Figure 1:**
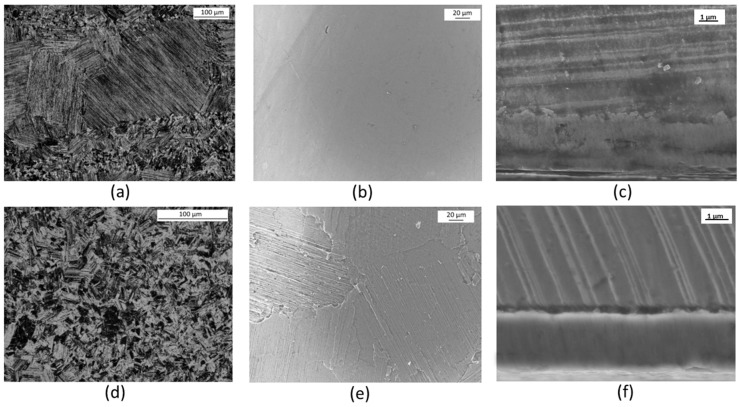
(**a**) Ti-Al intermetallic alloy, dual phase microstructure (optical microscope, etching by Kroll’s reagent); (**b**) coating surface of TiAlN1 specimen (SEM, InLens); (**c**) cross section of TiAlN1 sample (SEM InLens, etching with NH_4_OH:H_2_O_2_:H_2_O 1:2:5 volume ratio); (**d**) Ti-Al intermetallic alloy, colonies of gamma grains (optical microscope, etching by Kroll’s reagent); (**e**) coating surface of TiAlN2 specimen (SEM, InLens); and (**f**) cross section of TiAlN3 sample (SEM SEI, etching with NH_4_OH:H_2_O_2_:H_2_O 1:2:5 volume ratio).

The Ti-48Al-2Cr-2Nb intermetallic alloy, used as reference material in burner rig test and adopted as substrate for the HiPIMS deposition of TiAlN coatings, shows a typical duplex microstructure (Figure 1a,d). This microstructure is believed to ensure the best compromise of strength, ductility and fracture toughness [2]; such appropriate combination of properties is required for structural components of turbines.

The duplex microstructure is made of regions showing fine lamellar colonies and regions mainly containing equiaxed gamma grains. The lamellar structure consists of a mixture of alternate plates of γ phase (cubic TiAl) and plates of α_2_ (Ti_3_Al with hexagonal lattice and ordered structure). The dual phase microstructure is achieved by using a proper Al:Ti atomic ratio as well as by adopting a specifically designed thermal treatment. The lamellar structure provides creep resistance and fracture toughness while the dual phase structure, arising from the combination of lamellar regions with the gamma equiaxed grains, ensures the necessary ductility at room temperature. Chromium is added to improve ductility too and Nb enhances the oxidation resistance. The microscopic examination of the surface of the TiAlN coatings shows that they are continuous, flat and not porous (Figure 1b,e).

**Figure 2:**
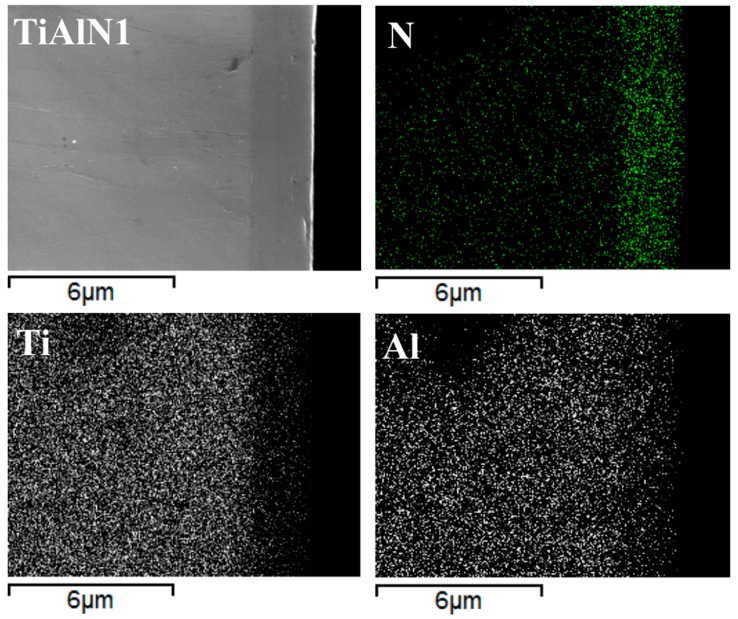
Cross section of TiAlN1 sample: coating morphology and nitrogen, titanium and aluminum concentration maps.

The coatings do not completely hide the microstructure of the substrate from microscopic observation. This is particularly evident in the case of sample TiAlN2 since the high energy metallic ions bombardment performed before the coating deposition etched the substrate surface adding evidence for the duplex microstructure (Figure 1e). The cross sections of the coated specimens (Figure 1c,f) allowed measuring their thicknesses, which were similar for the three coatings under investigation and almost uniform all over the sample surfaces (between 2 and 2.2 µm). Elemental maps recorded on the sample cross sections (Figure 2) showed that nitrogen is present within the coating, where the concentration of metallic elements (Ti and Al) is about the half of that observed in the TiAl alloy substrate. Chromium and niobium were not detected inside the coating, which means that any diffusion from the substrate towards the surface did not occur during HiPIMS deposition. 

After burner rig test, the morphology of the surface changed because of the growth of an oxide layer. This was well evident for sample TiAlN2 because the growing oxide film hid the intermetallic alloy microstructure. A microscopic inspection of the oxidized surfaces suggested that the white oxide film is not fully homogeneous. This behavior is in agreement with the pitting mechanism of corrosion reported in the literature for the initial corrosion step of TiAlN end TiN surface coatings [21]. Actually, it is very hard to determine accurately the thickness of the oxide layer using microscopy. Indeed, even if the specimen is mounted using a thermoset resin before cutting and polishing, the oxide film (a few tenths of nanometers thick) can suffer damage and partial removal from the sample cross section during the sample preparation for the microscopic examination. Nevertheless, the SEM-EDS analysis of the oxidized surfaces confirmed the formation of the oxide scale and allowed making comparisons between bare and coated specimens. Indeed, after burner rig test involving 40 cycles up to 950 °C the content of oxygen detected on the surface of the uncoated alloy was 51 at % while it ranged between 17 and 21 at % for the specimens carrying the TiAlN coatings. This outcome represents a first evidence of the coating effectiveness. 

**Figure 3:**
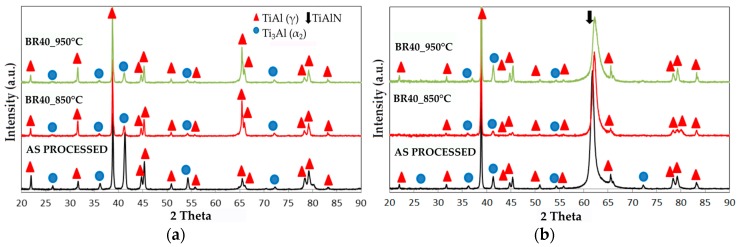
X-ray diffraction (XRD) spectra of samples surface before burner rig tests, after 40 cycles up to 850 °C and after 40 cycles up to 950 °C: (**a**) Ti-48Al-2Cr-2Nb alloy (substrate); and (**b**) TiAlN1 coating.

The XRD patterns detected on the surface of both the intermetallic alloy and the coated specimens are compared before and after oxidation in Figure 3. In this figure, only the patterns of uncoated substrate and coated TiAlN1 sample are reported since the XRD patterns of TiAlN2 and TiAlN3 specimens were similar.

The XRD spectrum of uncoated alloy shows the characteristic peaks of TiAl (γ) and Ti_3_Al (α_2_) phases, while only an additional intense peak, referring to a mixed nitride of Ti and Al phase, was detected at 2θ = 62.24° in the XRD patterns of the TiAlN coated specimens. The mixed nitride, which forms when Ti atoms in the TiN lattice (B1 NaCl-type) are substituted by Al atoms [38], can be indexed with the (AlTi)N phase, card number #58012 of the ICSD database [39]. The intensity of the (220) peak was dominantly high in collected spectra, meaning (220) preferential orientation. Very likely the other diffraction peaks (111), (200), (311) and (222) could not be observed in the XRD patterns because they were overlapped with the reflexes of γ and α_2_ phases or they were not enough intense to exceed the background. Moreover, it is well known that magnetron sputtering based technologies can cause coating texture. In particular, (220) texture was already reported in the literature [40,41,42]. The burner rig test carried out at 850 °C or at 950 °C did not result in the appearance of oxide peaks in the XRD patterns, probably because a very thin and not well crystallized oxide layer forms under these conditions. On the contrary, the (220) peak of TiAlN still remained in the XRD spectra detected after oxidation (Figure 3). Actually, the X-rays penetrates several microns below the sample surface and therefore both the peaks characteristics of the nitride coating and the intermetallic substrate can be seen in the relevant XRD pattern. XRD allows only crystalline phases to be detected and only if their amount inside the volume of material affected by the analysis exceeds the detection limits of this technique. The absence in the XRD patterns of oxidized samples of peaks belonging to oxides suggests that the amount of oxides and therefore the thickness of the oxide layer are quite small. On the contrary, XPS is currently widely used for investigating very this surface coatings since the probe enters the sample only for a few atomic layers and also amorphous phases give a response in photoelectron spectroscopy. Therefore, the XPS technique is well suited for investigating very thin surface layers, since XPS results refer to the external layer only without any disturbance given by the underlying material (substrate).

### 3.2. XPS Chemical Analysis

**Figure 4:**
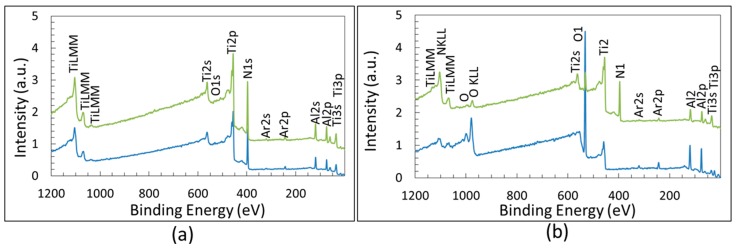
XPS survey of samples after surface cleaning: (**a**) TiAlN1 (blue) and TiAlN3 (green) as deposited; and (**b**) TiAlN1 (blue) and TiAlN3 (green) after 40 cycles at 950 °C in burner rig.

The XPS survey spectra for coated specimens before and after the burner rig test (which entails oxidation) are compared in Figure 4. This figure shows the survey spectra of sample TiAlN1 and TiAlN3, which are similar to those recorded for TiAlN2. Obviously a difference in the intensity of the peaks belonging to oxygen and nitrogen can be found depending on the relative amount of oxides and nitride within the zone affected by XPS analysis. The comparison of the survey spectra of oxidized samples TiAlN1 and TiAlN3 in Figure 4 shows that the intensity of the oxygen peak is higher and that of the nitrogen peak is lower for TiAlN1 with respect to TiAlN3. This outcome (as corroborated by results in Table 2) suggests that the oxide layer is thicker for TiAlN1 samples. The peaks corresponding to the binding energies (BEs) of Al2p, N1s, Ti2p and O1s (clearly visible only after burner rig test, see Figure 4b) are well evident and they were used for investigating more in detail the chemical bonds formed by these elements through the high resolution spectra. Peaks belonging to Nb3d and Cr2p were observed only in the survey spectrum of the uncoated Ti-Al intermetallic alloy, while the N1s peak obviously is not present in this case. Peak of C1s was observed only before the surface cleaning carried out by sputtering, which caused the appearance of the Ar2p signal instead. The high resolution spectra of Al2p, N1s, Ti2p and O1s were generally fitted by several peaks with the aim of achieving information about the chemical composition and bonding characters of surface films before and after burner rig test. In principle, the integral of the deconvoluted peaks can be used to estimate quantitatively the bond contents. Unfortunately, the tight overlapping of peaks belonging to different bonds, which occurs mainly after the formation of oxides, and some uncertainties in the literature about the binding energies made this estimation difficult to be done with accuracy. Nevertheless, significant semi-quantitative indications about the importance of the different bonds can be obtained by fitting and integrating the high-resolution (HR) spectra.

**Figure 5:**
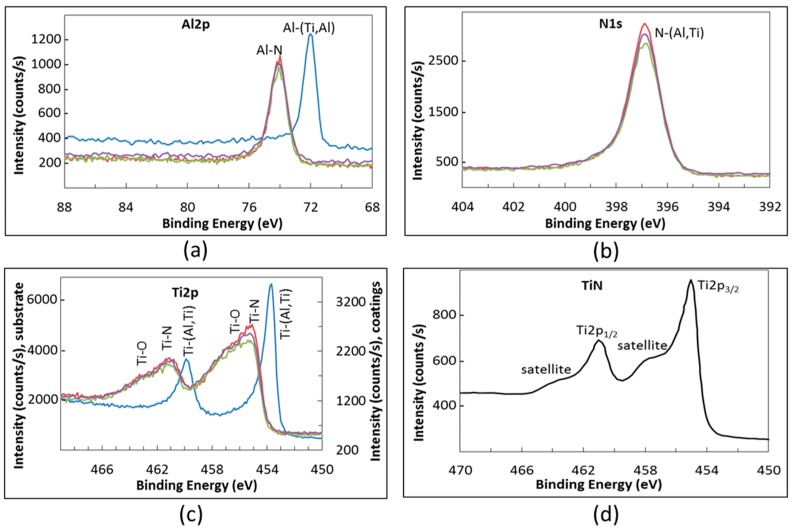
(**a**–**c**) XPS High resolution spectra of substrate and as-deposited TiAlN films: blue = uncoated intermetallic; red = TiAlN1; green = TiAlN2; and purple = TiAlN3; and (**d**) HR Ti pattern of pure TiN.

The HR spectra recorded for the as-processed specimens are shown in Figure 5. The Al2p HR spectrum of the uncoated intermetallic alloy (Figure 5a) showed a single peak at 71.9 eV which can be attributed to metallic bonds. Indeed, according to the literature, the BEs for Al-Al and Al-Ti are 72.8 eV [40] and 71.5 eV [43], respectively. The binding energy for Al-N bond is higher since the Al2p_3/2_ peak for this bond was observed in the range between 73.7 and 74.3 eV [40,41,42,43,44]. Shifts of the peak position very likely depend on the stoichiometry of the aluminum nitride. In the Al2p, HR spectrum of as-processed TiAlN coatings, we observed a peak at 74.2 eV in agreement with the literature data (Figure 5a). When some oxygen remained on the sample surface (in spite of the surface cleaning by sputtering) the fitting of the Al2p peak required a small contribution of a peak at 75.6 eV that can be attributed to Al-O bonds characteristic of alumina [40,44]. The N1s HR spectrum detected on coated samples confirmed the presence of the nitride (Figure 5b). The XPS literature spectra of TiAlN reported a nitrogen-(Ti,Al) peak ranging between 396.4 and 398.6 eV. This is consistent with the formation of both N-Ti and N-Al bonds that show peaks placed at 396.7–398.0 eV and at 396.8–398.6 eV, respectively [40,41,43]. The differences found by different authors regarding the N-Ti and N-Al BEs have been attributed to a variation of nitride stoichiometry as well as to oxygen contamination [42,45]. For all the specimens carrying TiAlN coatings, we found a main peak for N1s with its maximum at 396.9 eV.

The Ti2p HR spectrum of as processed specimens was more complex, because the spin-orbit splitting results in the formation of a doublet, consisting of Ti2p_3/2_ and Ti2p_1/2_ peaks. According to the literature, when Ti forms metallic bonds, two peaks at 453.7 eV and at 459.8 eV appear in the relevant XPS spectrum [46]. Actually, only this doublet was found in the spectrum of the uncoated TiAl alloy (Figure 5c). The titanium nitride spectra also show a spin orbit doublet. The Ti2p_3/2_ peak was previously observed in the BE range between 454.4 and 455.8 eV [40,41,42,43,44,45], while the component of the doublet Ti2p_1/2_ was detected at 460.2 eV [42] or at 461.1 eV [43]. 

Both these components of the doublet have weak satellites shifted with respect to the main peaks towards higher energies (as shown for TiN in Figure 5d). In addition, according to the literature, the partial oxidation of the nitride, giving Ti-N-O species, easily results in a shift of the Ti-N peaks towards higher energies [41,43,44,45]. In the Ti2p HR spectrum of coated specimens we always observed the doublet for Ti-N bonds at 455.1 eV and 461.0 eV (Figure 5c). The presence of oxygen as contaminant of the surface can make the Ti2p spectrum more complex and therefore also a peak belonging to Ti-O bonds must be used for the fitting. The effect of formation of oxides on the features of both Ti2p and O1s HR spectra is better discussed in the following analysis of XPS spectra of oxidized specimens.

**Figure 6:**
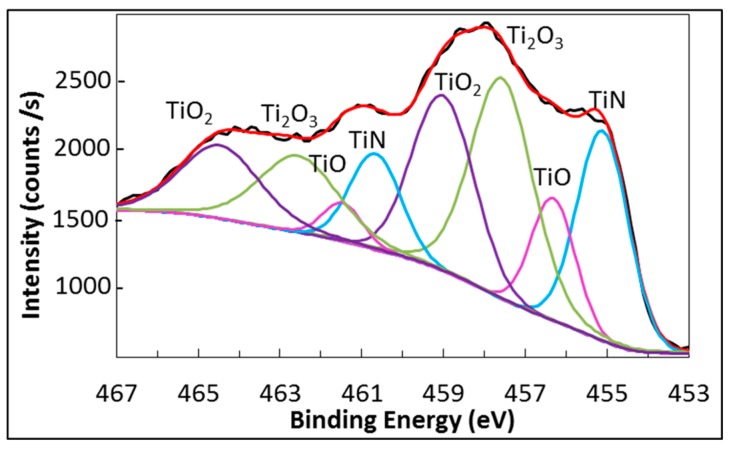
TiAlN1 after 40 burner rig cycles up to 850 °C: fitting of Ti2p peaks.

The oxidation of samples occurring during the burner rig tests made the Ti2p HR spectrum very complex since, in addition to Ti-N bond, two or three kinds of Ti-O bonds must be considered for the fitting. An example of fitting for Ti2p HR spectrum is shown in Figure 6, which refers to the spectrum observed for sample TiAlN1 after 40 cycles carried out in the burner rig apparatus up to 850 °C. This spectrum was fitted by four curves (represented by different colors in Figure 6) that correspond to four titanium chemical states. Each of these curves gives a contribution to fit both the Ti2p_3/2_ and the Ti2p_1/2_ peaks. In this case (after 40 cycles at 850 °C), the Ti2p_3/2_ peak can be deconvoluted in four (partially overlapped) peaks. The first one at 455.1 eV was assigned to Ti-N, as mentioned above, and the second one, observed at 456.0 eV, to Ti-O bond characteristic of TiO. The fitting also involved two additional peaks corresponding to binding energies ranging between 457.8 and 459.0 eV. Actually, according to the literature [47], the oxidation of titanium can cause the formation of the oxides TiO, Ti_2_O_3_ and TiO_2_ that gives rise to peaks (in the Ti2p_3/2_ spectrum) with progressively increasing binding energies. 

There is no agreement in the literature about the binding energy of Ti-O bonds in TiO since it was reported to be 456.2 eV [42], 455.1 eV [48] or 455.3 eV [49]. The Ti_2_O_3_ is believed to show a peak at 457.5–458.3 eV [44,48], which is superimposed to the peak characteristic of TiO_2_ placed in the range between 457.9 and 458.8 eV [40,41,42,43,44,45,50]. Because of the scattering of literature data about BE of Ti-O_2_ bond and the little information about XPS spectrum of Ti_2_O_3_, the assigning of the two peaks that we observed in the range between 457.5 and 459.0 eV was quite puzzling. Nevertheless, the formation of three oxides with different oxidation states for Ti is obvious (Figure 6). Similarly, the fitting of the Ti2p_1/2_ peak in Figure 6 can be done using four peaks at 461 eV, 461.8 eV, 463 eV and 464.6 eV, which we assigned to TiN, TiO, Ti_2_O_3_ and TiO_2_, respectively.

**Figure 7:**
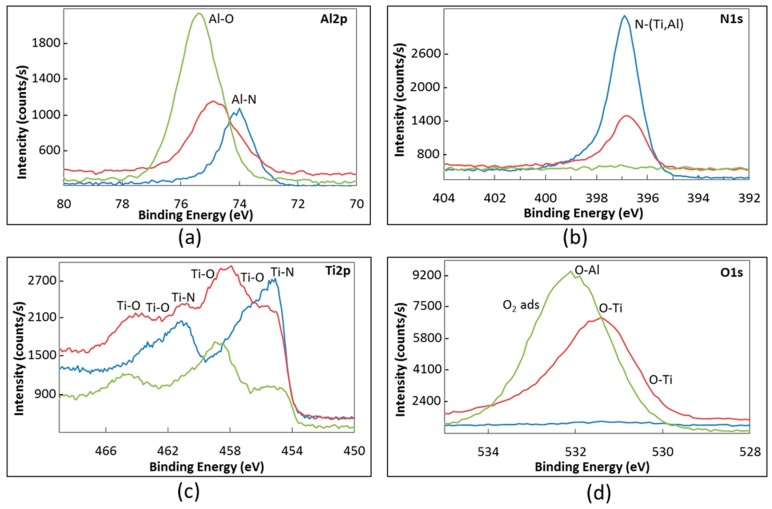
XPS high-resolution spectra of TiAlN1 sample (**a**–**d**): blue = as processed; red = after 40 burner rig cycles up to 850 °C; and green = after 40 burner rig cycles up to 950 °C.

According to the literature, the BE of O-Ti bonds also depends on the oxidation state of titanium. Indeed, the O-Ti^4+^ peak in the O1s HR spectrum was reported within the range 529.6–530.4 eV [40,42,43,44,45] while the O-Ti^2+^ peak was observed at 531.2 eV [48]. Finally, the binding energy ranging between 530.3 and 532.15 eV was attributed in the literature to O-Al bond [43,44,45,50]. In addition, oxygen adsorbed on the surface is believed to give a peak in the XPS spectrum at 531.8–533 eV [42,43,44]. On the basis of the literature data, the fitting of the O1s HR spectrum should involve three or four peaks. The progressive oxidation of samples occurring during the burner rig test resulted in a strong modification of the HR spectrum for Al2p, O1s and Ti2p, while mainly the intensity of the N1s peak in the relevant spectrum was affected by the test. These changes in the XPS analysis of the sample surface are depicted in Figure 7, which refers to sample TiAlN1 taken as an example.

Before oxidation, the Al-N peak dominates the Al2p spectrum. With the oxidation advancement the Al-O peak progressively grows, becoming the most important one after 40 burner rig cycles up to 950 °C (Figure 7a). The O1s spectra recorded on samples before and after cyclic oxidation at 850 °C and 950 °C are compared in Figure 7d, where the positions expected for the peaks of O-Ti^4+^, O-Ti^2+^, O-Al and adsorbed oxygen are also reported. It is clear that the intensity, the shape and the position of the peak in the O1s spectrum changes with the progress in oxidation. The contribution of the O-Al peak (at 532.2 eV) becomes dominant after 40 cycles at 950 °C, while after 40 cycles at 850 °C the contribution of the peaks of O-Ti bonds (at 531.2 eV and 530.3 eV) is also very important.

In the Ti2p spectrum, the peak related to Ti-N bond was the most important before the oxidation test, but its intensity progressively decreased with the oxidation advancement while several peaks relevant to the formation of several titanium oxides (as discussed above) progressively grew (Figure 7c). Finally, the N1s spectrum seemed not to be appreciably affected by the oxidation process. It consisted of a N-(Al,Ti) peak as long as nitrogen could be detected, while it disappeared in the XPS spectrum when the oxide scale became thick enough to hide the nitride coating (Figure 7b).

The outcomes of XPS analyses are summarized in Table 2 and Table 3, which show the chemical compositions (atomic percent) of the sample surface, the kind of chemical bonds detected and the corresponding chemical species observed; these species are listed according to their percentage from the most important component of the film surface to the less important one.

The survey XPS analysis of as-processed samples put in evidence that surface contamination due to oxygen and carbon occurred. The contamination of the surface by oxygen gave rise to the formation of a mixture of titanium oxide, aluminum oxide and adsorbed oxygen. Anyway, the sputter cleaning of the sample surface resulted in the almost complete disappearance of oxygen and carbon but argon (used for sputtering) contaminated the sample surface. After the cleaning sputtering the XPS analyses of the TiAl alloy and those of as-deposited specimens showed that the compositions of the coated specimens and the uncoated alloy were consistent with the nominal ones (Table 2). Actually, after the sputtering an atomic ratio between Ti and Al close to 1:1 was found on the surface of uncoated samples while for the coated samples the Ti:Al:N atomic ratio was consistent with the presence of a (Ti_0.5_Al_0.5_)N coating. After the burner rig test lasting 40 thermal cycles up to 850 °C, the oxygen percentage greatly increased on the surface. In every case (bare substrate and samples protected by the three nitride coatings), a surface layer consisting of aluminum and titanium oxides grew (Table 2). The oxide scale thickness seems greater for the bare TiAl alloy. 

In fact, after oxidation, every peak related to the metallic substrate disappeared in the spectrum of uncoated alloy while evidences of the TiAlN coating were still present within the spectrum of the coated specimens. The burner rig test carried out at higher temperature (namely 950 °C) obviously resulted in a major growth of the oxide scale, which mainly consisted of alumina and titanium dioxide (with some evidence of the presence of Ti^3+^ and Ti^2+^ oxide too). After this test at 950 °C the oxide layer was thinner in the case of TiAlN3 sample, since only in this case nitrogen and nitrides were still detected in large amount in the XPS surface spectrum. In every case there was not evidence of the presence of Nb and Cr within the oxide layer grown on the surface of the coated samples. Progressive sputtering of the sample surface allowed confirming the previous outcomes about the thickness of the oxide layer. The XPS analyses of coated samples (oxidized during burner rig test at 950 °C) were compared after different sputtering periods in Table 3. From these results, it is clear that the oxide layer grown on TiAlN3 sample is much thinner than those formed on both TiAlN1 and TiAlN2. Indeed, an 8 min long sputtering performed on TiAlN3 surface (in addition to the usual sputtering required for cleaning) was sufficient for greatly decreasing the oxygen percentage (2.9 at %), increasing the nitrogen one and, as a consequence, observing a higher intensity of the peak related to Ti-N bonds in the HR Ti2p spectrum. On the contrary, after a much longer sputtering period (20 min) carried out on the TiAlN1 and TiAlN2 samples, oxygen and oxides peaks were still dominating the XPS survey and HR spectra. In addition, the XPS spectrum recorded after 20 min of sputtering for TiAlN1 showed the presence of 10 at % of nitrogen too, while this did not happen in the case of TiAlN2. In this last case, a sputtering lasting even 50 min was not sufficient to put in evidence the peaks related to the nitride coating. Therefore, the oxidation kinetics for samples carrying TiAlN coatings with the same composition was found to be very different: the thinnest oxide layer growing on the TiAlN3 sample and the thickest one growing on the TiAlN2 specimen.

### 3.3. Scratch Tests and Wear Behavior

The scratching tests were used to assess the adhesion strength of the TiAlN coatings to the substrates for as deposited samples and after the burner rig test up to 850 °C (40 thermal cycles). 

**Figure 8:**
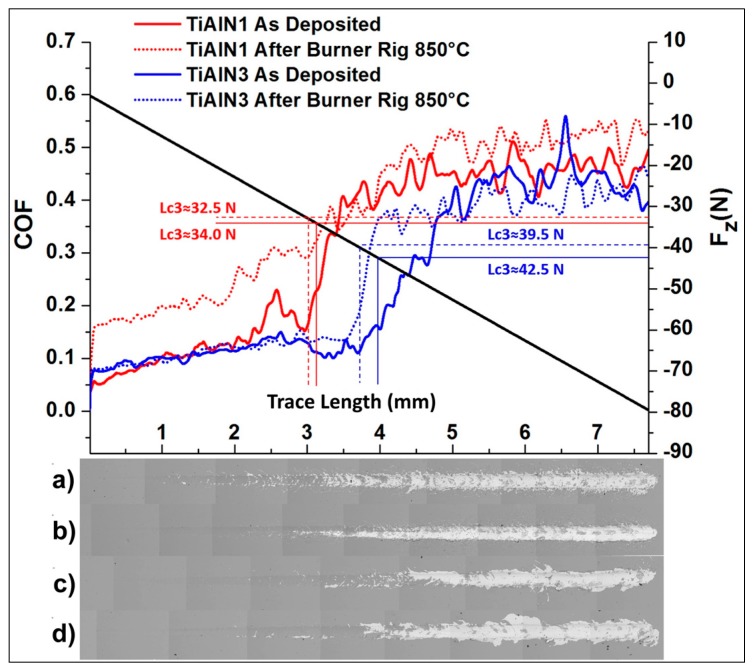
COF curves recorded during a single scratch test for the TiAlN1 sample and the TiAlN3 sample before and after burner rig (BR) tests (40 cycles up to 850 °C) are reported. SEM images of the associated scratch tracks are depicted too: (**a**) TiAlN1 as deposited; (**b**) TiAlN1 after BR; (**c**) TiAlN3 as deposited; and (**d**) TiAlN3 after BR. As an example, evaluated Lc3s are compared.

The film/substrate adhesion results are reported in Table 4, which summarizes the Lc3 critical load values, associated to the coating adhesion failures. The average value was obtained considering three different tests for each sample. The critical normal load could be detected when the film started to delaminate by the appearance of a discontinuity in the friction coefficient curve (Figure 8).

As reported in Table 4, the plasma etching treatment performed on TiAlN2 samples did not significantly affect the Lc3 critical load values. Instead, the plasma etching treatment followed by the deposition of a glue TiAl metal interlayer clearly improved the film/substrate adhesion properties for TiAlN3 samples [51]. The average Lc3 value increased from (34.0 ± 0.5) N for TiAlN1 sample to (42 ± 3) N for TiAlN3 sample. The burner rig thermal treatment did not weaken the adhesion of the film to the substrate. Indeed, the Lc3 load values after 40 cycles at 850 °C were not far from the ones detected on the as deposited samples.

In Figure 8, as an example, one scratch test output for the TiAlN1 sample as deposited and after the burner rig test and one for the TiAlN3 sample are shown. By carefully observing the scratch tracks of the as deposited films, it was found that there were evident conformal buckle cracks (semicircular and parallel to the leading edge of the moving stylus) and little delamination at the scratch track edges at applied loads much lower than Lc3. On the contrary, after the thermal shock treatment, the coatings could deform plastically up to Lc3 without obvious damages (Figure 8). According to some literature [52], it is possible to assume the heat treatment allowed internal stress relaxation. These results pointed out that the thermal treated films exhibited a higher toughness than the as deposited ones.

The wear behavior of uncoated and coated samples in the as processed state and after 40 cycles up to 850 °C are summarized in Table 4. As expected, the nitride coatings greatly increased the surface wear behavior [53]. Nevertheless, from XPS analyses, the formation of an oxide layer was detected after the burner rig test. This oxide layer was responsible for changes in surface chemical composition and, outwardly, in surface mechanical properties of the tested samples. As a result, also the interaction between sliding bodies was affected. The oxide layer influenced the wear behavior of substrate and coated samples in different ways. After the burner rig test the substrate showed an almost unchanged friction coefficient but a higher wear rate. For coated samples, the friction coefficients appreciably decreased and this resulted in lower wear rates (Table 4). Therefore, burner rig test further increased the differences between substrate and coated samples.

**Figure 9:**
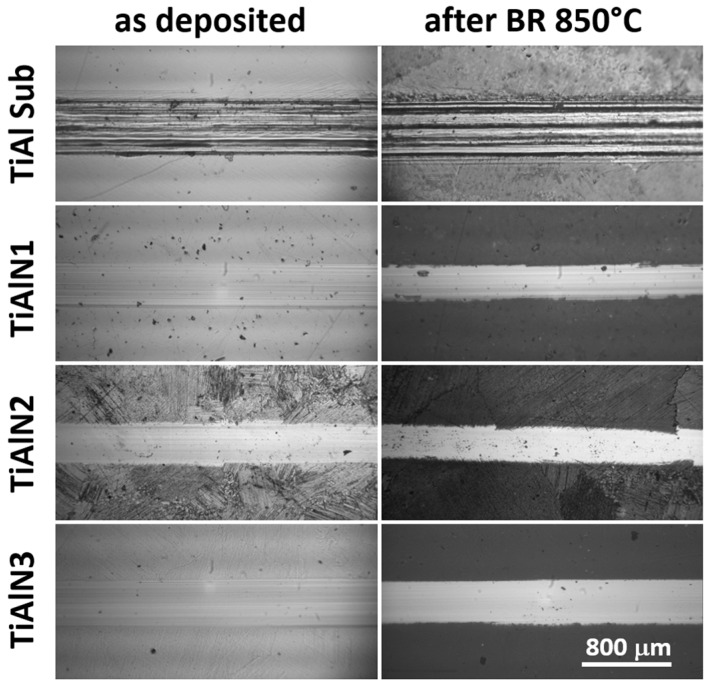
Comparison of wear track optical microscopy images: on the left as deposited samples are shown, on the right samples after 40 cycles up to 850 °C thermal treatment are presented.

Inside the wear tracks, optical micrographs showed some micro scratches, which might rise from coatings and oxide layer debris that were detached, trapped under the sliding ball, and dragged along the film surface. After the burner rig test, the coated samples demonstrated a more homogeneous wear with a slighter track width and less evident scratches (Figure 9). 

On the contrary, for the substrate scratches after thermal cycling were deeper, thus suggesting that debris played a major role in its wear process. 

As regards coated samples, since produced nitride coatings have a sufficient adhesion strength, the wear mechanism should be chiefly ruled by the surface interaction of tribologically coupled materials [54,55]. In addition, the slightly improved toughness observed in the scratch tests after testing could favor wear resistance. Finally, for nitride coatings that have a very high hardness (>30 GPa according to our measurements), oxides could act as lubricants, thus providing lower friction coefficients [54].

## 4. Discussion

During the burner rig tests, the specimens underwent very severe thermal shocks. For each thermal cycle the heating and the cooling rates were 141 °C/min and 185 °C/min respectively. Under these conditions evident thermal stresses should occur, owing to the different thermal expansion coefficients of the intermetallic alloy 10 × 10^−6^ K^−1^ (our measurement) and of the TiAlN coating (7.5 × 10^−6^ K^−1^ [14]). Moreover, it is believed that additional residual stresses occur inside a TiAlN coating because of the HiPIMS deposition method [19,20,40]. Nevertheless, in this study, the microstructure of the coated specimens after burner rig tests never showed evidence of failure or loss of adhesion between the intermetallic substrate and the nitride coating. This successful result was confirmed by scratch tests. Indeed, the coating delamination loads (Lc3), which were measured just after film depositions, were very similar to the Lc3 values observed after 40 cycles at 850 °C. In short, thermal cycling seems not to have affected film/substrate adhesion for every processing mode adopted for the coating deposition. 

On the other hand, the experimental procedure chosen for the coating deposition greatly affected the kinetics of coating oxidation and showed some slight influence on the coating adhesion too.

The substrate HiPIMS pretreatment (samples TiAlN2 and TiAlN3) utilized pulses with peak power densities of about 500 W·cm^−2^ applied to the TiAl target. This aggressive plasma etching step chosen for TiAlN2 (60 min) significantly altered the surface morphology and roughness of the substrate Ra ~ 130 nm vs. Ra ~ 10 nm for TiAlN1. It is recognized that during PVD film growth, an increased substrate surface roughness results in a more defective coverage because of shadowing effects [56,57]. HiPIMS films, however, are extremely dense and smooth thanks to the high energy of the arrival particles, ad-atom mobility and nucleation density increase. The atomic shadowing effect for HiPIMS films during the coating growth is significantly reduced but is still present when the substrate surface roughness is high. Consequently, the grown TiAlN2 coating was probably more defective and highly stressed. Moreover, the surface roughness of the sputtered TiAlN coating increased with the roughness of the TiAl substrate. This resulted in a greater exposed surface area, which favored the oxidation process (as confirmed by XPS analyses).

In the TiAlN3 case, a soft plasma etching step (10 min) was followed by the introduction of a 300 nm thick TiAl metal interlayer, which resulted in a clearly defined and sharp interface with a coherent TiAlN layer growth. Ion etching pretreatment plus a metal interlayer deposition positively influenced properties such as adhesion (as the deposited TiAlN3 has the maximum Lc3 = 42 ± 3 N) and oxidation performance (confirmed by XPS analyses). The metal interlayer has a higher ability to deform plastically if compared to the nitride coating. For this reason when deforming during the adhesion test, the high local stresses within the film induced by the scratching stylus were reduced and the measured adhesion value was enhanced. 

Obviously, the thickness of the grown surface oxide films increased with the oxidation temperature, but it still remained very thin in the experimental conditions adopted in the present work. For this reason, nitrogen and nitrides were always present in the XPS surface spectra of coated samples after 40 cycles at 850 °C. Instead, after 40 cycles up to 950 °C, the nitrogen quantity greatly decreased for TiAlN1, totally disappeared for the TiAlN2 sample, and remained quite high in the TiAlN3 specimen case. The repetition of XPS analysis after progressive surface sputtering confirmed that a thinner oxide film formed on the surface of the TiAlN3 coated set of samples. These outcomes highlight that the processing method selected for the coating deposition greatly affected the oxide layer thickness. The soft plasma etching pretreatment process probably cleaned and activated the sample TiAlN3 substrate surface while maintaining a low surface roughness (Ra ~ 30 nm) and promoting a reduced number of coating flaws. Moreover, HiPIMS is known to produce a defect free closed microstructure and to improve film density: this technology is able to affect oxidation performance by blocking oxygen diffusion [51].

The morphology of the oxidized sample surface suggested that in every case the oxidation during the initial stage did not happen homogeneously for both the uncoated and coated intermetallic alloy. This is not surprising at all in the case of the dual phase intermetallic alloy since aluminum diffusion towards the surface is believed to control the oxidation rate. In addition, the diffusion of this element occurs more quickly in γ phase than in α_2_ phase (aluminum diffusion coefficients at 650 °C are equal to 1 × 10^−21^ m^2^·s^−1^ and 1 × 10^−23^ m^2^·s^−1^ respectively) [50]. On the other hand, it was also reported that a corrosive attack causes damage by pitting of TiAlN coatings [21].

As expected the oxidation of these specimens resulted in the formation of alumina and several titanium oxides with different oxidation states of the titanium (Ti^2+^, Ti^3+^ and Ti^4+^). Among the titanium oxides, TiO_2_ was found to be the predominant one. The atomic ratio between aluminum and titanium in the XPS spectrum of the uncoated alloy, either before or after oxidation, was always close to 1:1, which means that the presence of these two elements in the oxide film reflects their presence in the intermetallic alloy. The Al:Ti ratio still remained close to 1:1 after 40 cycles even for the TiAlN3 sample, which showed only a limited growth of the oxide layer. In contrast, for the TiAlN1 and TiAlN2 the comparison between the XPS spectra after burner rig testing at 850 °C and 950 °C showed that the Al:Ti ratio in the oxide scale increased with the oxidation temperature and the growth of the oxide layer. This feature suggests that the oxide film can progressively acquire a passivating character as the oxidation process advances only in the case of coated samples.

In this work, the thermal stability was also considered in relation to the exploitation of TiAlN coatings for oxidation protection. TiAlN is a supersaturated solid solution with a cubic crystal lattice similar to that of TiN. This solution is believed to be metastable, since annealing can result in its spinodal decomposition with the formation of cubic Ti-rich and Al-rich domains and the latter further transform into an AlN wurtzite-like structure. The decomposition is favored by a high Al:Ti ratio in the nitride and high temperature exposure [15,16,17]. For instance, according to the literature, the Ti_0.54_Al_0.46_N phase is stable up to 900 °C, while it starts to undergo decomposition from 1000 °C and shows a great instability over 1200 °C. Stability depends on the synthesis method, too. In the present work, no evidence of decomposition was found even after exposure of the TiAlN coatings to temperatures of up to 950 °C for quite a long period (forty burner rig cycles entailed isothermal steps at 950 °C lasting 2 h in total).

## 5. Conclusions 

TiAlN films were deposited by reactive High Power Impulse Magnetron Sputtering and different conditions were adopted for the preparation of the surface of the TiAl intermetallic substrates. Oxidation resistance and coating adhesion, which are among the major requirements for environmental barrier coatings of turbine engine components, were found to be very good for every coating under investigation. 

All of the TiAlN coatings significantly increased the oxidation resistance over that of the Ti-48Al-2Cr-2Nb substrate. In particular, a coating obtained by combining a soft HiPIMS plasma etching and the deposition of a TiAl interlayer before the processing of a TiAlN film resulted in a greater reduction of the oxidation rate. This improved performance was attributed to the growth of a coherent dense TiAlN film with a reduced flaw concentration.

The oxide layers grown on coated samples consisted in a mixture of alumina and several titanium oxides, with a ratio between the amounts of alumina and titanium oxides that progressively increases with the growth of the surface layer. This change of oxide layer composition should result in the progressive increase of its passivating capability.

The coating/substrate adhesion was roughly unchanged or even improved after the burner rig tests because of the stress relaxation phenomenon.

The TiAlN barrier deposited according to the HiPIMS method displayed very good thermal stability, as any evidence of spinodal decomposition was not observed after testing the specimens at temperatures up to 950 °C for several hours. 

The HiPMS technique provided protective coatings for a Ti-Al intermetallic substrate, which proved to be effective at temperatures above those presently required for industrial applications.

## Figures and Tables

**Table 1:** HiPIMS pretreatment and working parameters set for TiAlN coating deposition.

Deposition Steps	Woking Parameters	TiAlN1	TiAlN2	TiAlN3
**Substrate Pretreatment**	Ultrasound cleaning	yes	yes	yes
	HiPIMS plasma etching (min)	0	60	10
	TiAl Interlayer	no	no	yes
**Film Deposition Parameters**	Mean cathode power (W)	1000		
	Mean cathode power density (W/cm^2^)	12.5		
	Pulse length (μs)	25		
	Frequency (Hz)	500		
	Gas	Ar + N_2_ (50%)		
	Pressure (mbar)	1 × 10^−2^		
	Substrate bias voltage (V)	−50		
	Duration (min)	180		

**Table 2:** XPS surface analysis of specimens before and after burner rig test (40 cycles up to 850 °C and 950 °C).

Sample	O1s (at %)	O Chemical Bonds	Al2p (at %)	Al Chemical Bonds and Species	Ti2p (at %)	Ti chemical Bonds and Species	N1s (at %)	N chemical Bonds and Species	Others (at %)
TiAl alloy	0.8	O-(Al,Ti)	46.8	Al-M	43.3	Ti-M; (Ti-O)	0	–	Nb = 4.5 Cr = 4.7
TiAlN1	1.7	O-(Al,Ti)	23.7	Al-N; Al-O (AlN; Al_2_O_3_)	22.7	Ti-N; Ti-O (TiN; TiO)	50.1	N-(Al,Ti) (TiAlN)	Ar = bal.
TiAlN2	0.7	O-(Al,Ti)	21.7	Al-N; (Al-O) (AlN; Al_2_O_3_)	23.9	Ti-N; (Ti-O) (TiN; TiO)	52.2	N-(Al,Ti) (TiAlN)	Ar = bal.
TiAlN3	0.7	O-(Al,Ti)	24.0	Al-N; (Al-O) (AlN; Al_2_O_3_)	22.9	Ti-N; (Ti-O) (TiN; TiO)	50.7	N-(Al,Ti) (TiAlN)	Ar = bal.
TiAl alloy tested at 850 °C	63.4	O-Ti; O-Al; (C=O; ads O_2_)	17.2	Al-O (Al_2_O_3_)	15.7	Ti-O (TiO_2_; TiO)	–	–	Nb = 0.7 Cr = 1.4 Ar, C = bal.
TiAlN1 tested at 850 °C	52.5	O-Ti; 0-Al; (ads O_2_)	16.7	Al-O (Al_2_O_3_)	18.4	Ti-O; Ti-N (TiO_2_ + Ti_2_O_3_; TiN; TiO)	12.0	N-(Al,Ti) (TiAlN)	Ar = bal.
TiAlN2 tested at 850 °C	51.7	O-Ti; 0-Al; (ads O_2_)	17.8	Al-O (Al_2_O_3_)	18.5	Ti-O; Ti-N (TiO_2_ + Ti_2_O_3_; TiN; TiO)	12.0	N-(Al,Ti) (TiAlN)	–
TiAlN3 tested at 850 °C	7.4	O-Ti; 0-Al	21.8	Al-N; Al-O (AlN; Al_2_O_3_)	22.3	Ti-N; Ti-O (TiN; TiO)	46.2	N-(Al,Ti) (TiAlN)	Ar = bal.
TiAl alloy tested at 950 °C	67.6	O-Ti; O-Al	13.7	Al-O (Al_2_O_3_)	17.8	Ti-O (TiO_2_ + Ti_2_O_3_; TiO)	–	–	–
TiAlN1 tested at 950 °C	62.2	O-Ti; O-Al	29.4	Al-O (Al_2_O_3_)	7.0	Ti-O; Ti-N (TiO_2_ + Ti_2_O_3_; TiO; TiN)	1.4	N-(Al,Ti) (TiAlN)	–
TiAlN3 tested at 950 °C	16.1	O-Ti; O-Al	21	Al-O; Al-N (Al_2_O_3_; AlN)	21.8	Ti-N; Ti-O (TiN; TiO_2_ + Ti_2_O_3_; TiO)	41.1	N-(Al,Ti) (TiAlN)	–

**Table 3:** XPS surface analysis after 40 cycles up to 950 °C, comparison after different sputtering time.

Sample	O1s (at %)	O Chemical Bonds	Al2p (at %)	Al Chemical Bonds and Species	Ti2p (at %)	Ti Chemical Bonds and Species	N1s (at %)	N Chemical Bonds and Species
TiAlN1 as prepared	62.2	O-Ti; O-Al	29.4	Al-O (Al_2_O_3_)	7.0	Ti-O; Ti-N (TiO_2_ + Ti_2_O_3_; TiO; TiN)	1.4	N-(Al,Ti) (TiAlN)
TiAlN1 as tested, after 20 min of surface sputtering	52.8	O-Ti; O-Al	15	Al-O (Al_2_O_3_)	21	Ti-O; Ti-N (TiO_2_ + Ti_2_O_3_; TiN; TiO)	10	N-(Al,Ti) (TiAlN)
TiAlN2 as prepared	62.3	O-Ti; O-Al	31.9	Al-O (Al_2_O_3_)	4.4	Ti-O (TiO_2_ (+Ti_2_O_3_); (TiO)	–	–
TiAlN2 as tested, after 20 min of surface sputtering	64.1	O-Ti; O-Al	25.2	Al-O (Al_2_O_3_)	10.7	Ti-O (TiO_2_ +Ti_2_O_3_); (TiO)	–	–
TiAlN3 as prepared	16.1	O-Ti; O-Al	21	Al-O; Al-N (Al_2_O_3_; AlN)	21.8	Ti-N; Ti-O (TiN; TiO_2_ + Ti_2_O_3_; TiO)	41.1	N-(Al,Ti) (TiAlN)
TiAlN3 as tested, after 8 min of surface sputtering	2.9	O-Ti; O-Al	22.7	Al-N; Al-O (AlN; Al_2_O_3_)	23.5	Ti-N; Ti-O (TiN; TiO_2_ + Ti_2_O_3_; TiO)	50.9	N-(Al,Ti) (TiAlN)

**Table 4:** TiAlN films/substrates adhesion and wear behavior results.

Sample	Critical Load Lc_3_ (N)		Wear Rate mm^3^/(N∙m)		COF	
	As Deposited	After 40 BR Cycles up to 850 °C	As Deposited	After 40 BR Cycles up to 850 °C	As Deposited	After 40 BR Cycles up to 850 °C
Substrate	–	–	218 ± 19	299 ± 63	0.60	0.56
TiAlN1	−34.0 ± 0.5	−31 ± 1	37 ± 3	29 ± 6	0.95	0.58
TiAlN2	−35 ± 2	−38 ± 2	39 ± 7	31 ± 5	0.84	0.67
TiAlN3	−42 ± 3	−38 ± 2	37 ± 7	34 ± 8	0.84	0.69
